# Effect of Oral Beta-Hydroxy-Beta-Methylbutyrate (HMB) Supplementation on Physical Performance in Healthy Old Women Over 65 Years: An Open Label Randomized Controlled Trial

**DOI:** 10.1371/journal.pone.0141757

**Published:** 2015-11-03

**Authors:** Linda Berton, Giulia Bano, Sara Carraro, Nicola Veronese, Simona Pizzato, Francesco Bolzetta, Marina De Rui, Elena Valmorbida, Irene De Ronch, Egle Perissinotto, Alessandra Coin, Enzo Manzato, Giuseppe Sergi

**Affiliations:** 1 University of Padova, Department of Medicine-DIMED, Geriatrics Division, Via Giustiniani 2, 35128 Padova, Italy; 2 University of Padova, Department of Cardiac, Thoracic and Vascular Sciences, Biostatistics, Epidemiology and Public Health Unit, Via Loredan 18, 35128 Padova, Italy; Weill Cornell Medical College Qatar, QATAR

## Abstract

Although older people are particularly liable to sarcopenia, limited research is available on beta-hydroxy-beta-methylbutyrate (HMB) supplementation in this population, particularly in healthy subjects. In this parallel-group, randomized, controlled, open-label trial, we aimed to evaluate whether an oral supplement containing 1.5 g of calcium HMB for 8 weeks could improve physical performance and muscle strength parameters in a group of community-dwelling healthy older women. Eighty healthy women attending a twice-weekly mild fitness program were divided into two equal groups of 40, and 32 of the treated women and 33 control completed the study. We considered a change in the Short Physical Performance Battery (SPPB) score as the primary outcome and changes in the peak torque (PT) isometric and isokinetic strength of the lower limbs, 6-minute walking test (6MWT), handgrip strength and endurance as secondary outcomes. Body composition was assessed with dual-energy X-ray absorptiometry (DXA) and peripheral quantitative computerized tomography (pQCT). The mean difference between the two groups on pre-post change were finally calculated (delta) for each outcome. After 8 weeks, there were no significant differences between the groups’ SPPB, handgrip strength or DXA parameters. The group treated with HMB scored significantly better than the control group for PT isokinetic flexion (delta = 1.56±1.56 Nm; *p* = 0.03) and extension (delta = 3.32±2.61 Nm; *p* = 0.03), PT isometric strength (delta = 9.74±3.90 Nm; *p* = 0.02), 6MWT (delta = 7.67±8.29 m; *p* = 0.04), handgrip endurance (delta = 21.41±16.28 s; *p* = 0.02), and muscle density assessed with pQCT. No serious adverse effects were reported in either group. In conclusion, a nutritional supplement containing 1.5 g of calcium HMB for 8 weeks in healthy elderly women had no significant effects on SPPB, but did significantly improve several muscle strength and physical performance parameters.

**Trial Registration:**

ClinicalTrials.gov NCT02118181

## Introduction

The loss of muscle mass with aging, to the point of developing sarcopenia, is being recognized as a major health concern linked with declining physical function, lower quality of life and higher mortality. [1–3] The issue of sarcopenia also seems to be particularly relevant to older women, who on average have lower amounts of muscle mass than men, and twice the rate of decline in strength. [4]

Among the interventions that might delay progression to sarcopenia, dietary supplementation seems to be worth investigating. Beta-hydroxy-beta-methylbutyrate (HMB) has recently been considered for its multiple muscle-sparing properties: it enhances whole-body protein synthesis, increases collagen synthesis, inhibits protein degradation, and increases cholesterol synthesis in the muscle cell membrane. [5] HMB supplementation reportedly has positive effects on several conditions characterized by severe muscle mass loss, such as end-stage congestive heart failure, cancer and chronic renal disease. [5–9]

Although older people are particularly liable to sarcopenia, limited research is available on HMB supplementation in this age group. [5] Some studies on the elderly have shown a substantial positive effect of HMB supplementation on body composition and physical performance parameters, [9] but they were often based on analyses that did not distinguish between the genders, they focused mainly on patients with wasting diseases, and body composition measurements or physical performance parameters were often lacking. [10–14]

In the light of the previous literature, we hypothesized that HMB supplementation could improve physical performance and muscle function in active older women too. The aim of our study was therefore to evaluate whether an oral supplement containing 1.5 g of calcium HMB for 8 weeks could improve physical performance, muscle strength and body composition parameters in a group of healthy elderly women attending a twice-weekly mild fitness program.

## Materials and Methods

### Participants

This study was conducted at the Geriatrics Department of Padova University between 01 February and 30 June 2014. Women over 65 years of age were recruited on a voluntary basis from among older people attending a twice-weekly mild fitness program at public gyms in Padova. This training mainly consisted of aerobic exercises designed to improve speed of muscle contraction, and only a small part of it was dedicated to resistance exercises, essentially to improve handgrip strength.

Candidates were excluded if they showed signs of renal failure, chronic or acute infection, a history or evidence of malignancy in the past 5 years (except for non-melanoma skin neoplasia), significant cardiovascular or pulmonary diseases, uncontrolled metabolic diseases (diabetes, anemia or thyroid disease), or electrolyte abnormalities, or if they were already using dietary supplements other than cholecalciferol. During a screening visit, their healthy condition was ascertained by trained medical personnel based on their medical history, a clinical examination, and routine biochemical tests (e.g. glycemia, renal and liver function tests, proteins, electrolytes, and a complete blood count). During the follow-up, all participants continued their fitness program and their attendance was monitored and recorded twice a week.

**Fig 1:**
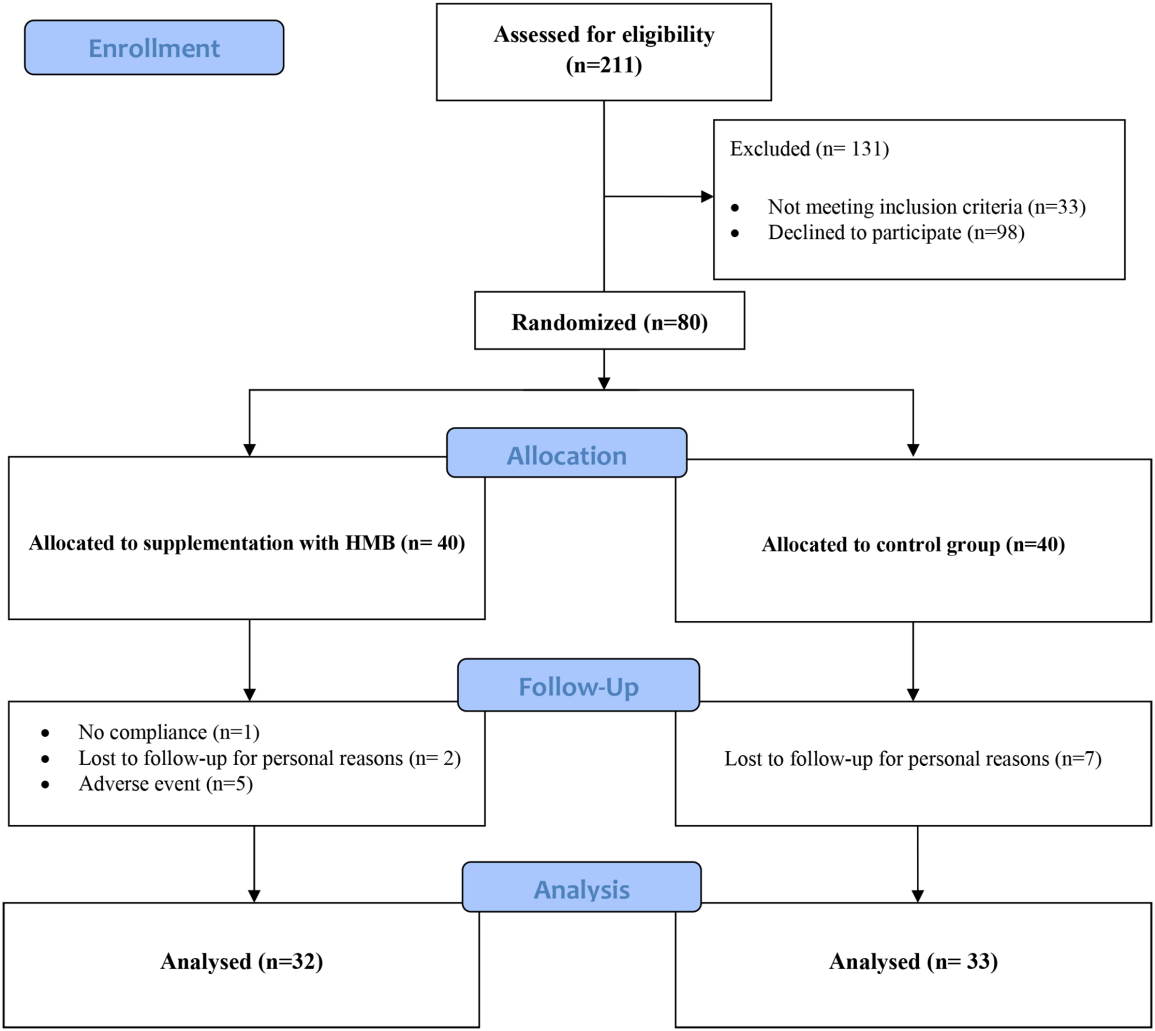
Consort diagram indicating sample sizes at each stage during the study.

The study was designed in accordance with the Helsinki Declaration and approved by the Ethical Committee (IRB) of Padova on 12 December 2013 (S1 and S2 Files). All participants were fully informed about the nature, purpose, procedures and risks of the study, and gave their written informed consent, which was recorded in their charts. This trial was registered at Clinical Trials Gov (website: https://clinicaltrials.gov/ct2/show/NCT02118181?term=hmb&rank=2; Clinical Trial Identification Number: NCT02118181) after the participants’ enrolment had begun due to technical issues (problems with Clinical Trials Gov login). The authors confirm that all ongoing and related trials for this drug/intervention have been registered. The study complies with the CONSORT Statement for randomized trials as shown in Fig 1 and S1 Table. [15]

### Outcome measures

All tests were performed at the baseline and again at the follow-up.

The primary outcome to measure was a change in the Short Physical Performance Battery (SPPB) score. [16] The SPPB score was obtained from three objective physical function tests, i.e. 4-meter walking time, repeated chair stands, and standing balance in increasingly challenging positions. Walking time was recorded as the best performance achieved in two walks at the participant’s usual pace along a corridor 4 m long. For the chair stands test, participants were asked to rise 5 times from a seated position as quickly as possible with their hands folded across their chest. For the standing balance tests, participants were asked to stand in 3 increasingly difficult positions (with their feet side by side, then in semi-tandem and full tandem positions) for 10 s each. Each test was scored from 0 (worst) to 4 (best), and the scores for all three tests were combined to obtain a composite score of 0 to 12, higher scores reflecting better physical function.

The secondary outcome measures concerned muscle strength and physical performance:

1. isometric knee extension torque and isokinetic (flexion and extension) strength were tested on the dominant side using a dynamometer chair (Easytech s.r.l., Florence, Italy). Participants were positioned upright with straps to fix their hips to the chair. Participants were asked to reach their maximal voluntary contraction for each of the three measurements, then to stop contracting 3 to 5 s after reaching their maximum effort. Each measurement was repeated 3 times and patients rested for 3 min between trials. The highest peak torque (PT) was used for the analysis.[17] The coefficient of variation (CV) for double determinations in 20 women was 7.5% for isometric and 7.7% for isokinetic strength. Previously-ascertained intraclass correlation coefficients (ICC), standard errors of measurement (SEM), and minimum differences needed to be considered real (MD) for isokinetic strength measured in 20 women (aged between 65 and 85 years) by three different clinicians were: ICC = 0.92; SEM ±5.9 Nm; and MD = 16.3 Nm [18];
2. 6-minute walking test (6MWT). Participants were asked to walk at their usual pace for 6 minutes, and the distance they covered was recorded in meters. [19] For this item, the ICC, SEM and MD previously ascertained in 10 women (aged between 65 and 74 years) by three different clinicians were: ICC = 0.94, SEM ±4.3 m, MD = 11.9 m;
3. handgrip strength was measured on the dominant side using DynEx electronic hand dynamometers (Ohio, USA). Participants were seated in a standard armchair with their shoulder adducted and neutrally rotated, their elbow flexed at 90°, and their forearm and wrist in a natural position. They were asked to grip the dynamometer, progressing smoothly up to their maximal strength in response to a voice command, without any wrenching or jerking motion. Three measurements were obtained with a 1 min rest between trials and the highest measurement was used in our analyses. [17] Handgrip endurance was tested by asking participants to maintain half of their maximal voluntary contraction for as long as they could, and the time was recorded in seconds using a stop watch. [20] The CV for double determinations was 3.3% for strength and 10.7% for endurance. For these items, the ICC, SEM and MD previously ascertained in 10 women (aged between 65 and 82 years) by three different observers were: ICC = 0.90, SEM ±1.5 kg, and MD = 4.3 kg for handgrip strength; and ICC = 0.93, SEM ±7.5 s, and MD = 20.8 s for handgrip endurance.

Body composition was assessed using:

1. dual-energy X-ray absorptiometry (DXA): body weight and height were measured by trained staff, and body composition was assessed using DXA with fan beam technology (Hologic Discovery A). Abdominal fat mass was investigated as a parameter of fat mass distribution because a previous study had shown that HMB could improve this parameter in older subjects. [21] The Appendicular Skeletal Muscle Mass Index (ASMMI), i.e. the ratio of appendicular skeletal muscle mass to height in kg/m^2^, and fat-free mass (FFM) were considered as indicators of muscle mass.[22] For the ASMMI, the ICC, SEM and MD previously-ascertained by three different physicians in 20 women aged between 65 and 85 years were: ICC = 0.95, SEM ±0.02 kg, and MD = 0.06 kg.
2. peripheral quantitative computerized tomography (pQCT) was performed on the dominant forearm and right tibia using the Norland/Stratec XCT-3000 scanner (Stratec Medizintechnik GmbH, Pforzheim, Germany), adopting a standardized patient positioning and scanning protocol. Forearm length was measured from the olecranon to the ulna styloid process, and tibia length from the medial malleolus to the medial condyle. A pQCT scout view was obtained to establish an anatomical reference line bisecting the medial edge of the end of the distal radius or tibia. Starting from this reference line, scans were obtained at sites 4% and 66% along the length of the shaft for the radius, and at sites 4%, 14%, 38%, and 66% along the length of the tibia. The following parameters regarding muscle and fat mass were considered: muscle density (mg/cm^3^), muscle and fat areas (mm^2^), and the ratio of fat to muscle (%). Muscle and fat areas were calculated at the sites 66% along the length of the tibia and radius. The muscle cross-sectional area included the areas of blood vessels, tendons and ligaments because they have the same attenuation coefficients as muscle; the fat mass area included both extra- and intramuscular fat. [23] The ICC, SEM and MD previously ascertained for radial muscle area by three different clinicians in 5 women aged between 65 and 70 years were: ICC = 0.85, SEM ±155.2 mm^2^, and MD = 428.4 mm^2^.

Physical activity levels were investigated using the Physical Activity Scale for the Elderly (PASE)—a scale validated for use in elderly adults—over the course of one week; higher scores indicated more time spent on physical activities. [24]

At the baseline, a trained dietician conducted a dietary assessment using a modified method based on recording the estimated food intake over 3 days and a questionnaire on the frequency with which participants usually ate certain foods, taking the previous month for reference. [25,26] The usual food intake was converted into macronutrients and micronutrients using a national food composition table. [27] The dietician asked both groups to avoid modifying their usual diet during the study.

### Randomization, intervention and allocation

Participants were randomly assigned to one of two experimental groups using a computer-generated sequence of 80 non-unique, unsorted numbers ranging from 1 to 2 and representing the groups. The main investigator (LB) and the research coordinator (NV) kept the allocation sequence confidential and assigned the women to one or other group.

Participants in the treatment group received 8 weeks of supplementation with a 220 ml drink containing 1.5 g of calcium HMB (Ensure Plus Advance^™^; Abbott Nutrition), while the control group received no treatment or placebo. The bottles (containing the daily doses of supplement) were contained in cartons (a month’s supply) and given to participants at the baseline and half-way through the study. Participants were encouraged to return the empty bottles after 4 weeks and at the follow-up visit. The supplement was to be taken in the morning during breakfast. The participants in the control group were encouraged not to take any nutritional supplements containing HMB or other substances/drugs capable of improving physical performance during the trial.

Weekly phone calls were made by the research coordinator (NV) to record the treated group’s compliance with the treatment, any adverse events, whether any drugs or supplements were taken by the control group, and both groups’ gym attendance for the fitness program. These details were also confirmed at face-to-face interviews after 4 weeks.

The two groups were followed up by a team of physicians and technicians who conducted physical and body composition tests at the baseline and again after 8 weeks. The same professionals performed the same tests at both time points. To ensure the success of the masking procedure, participants were instructed not to tell the people performing the tests whether they belonged to the treatment or the control group.

### Sample size and statistical analyses

The required sample size was calculated from the difference of 1 point in total SPPB scores between the treated and control groups after 8 weeks. With a standard deviation of 1.51 points (identified as clinically significant in a previous study conducted on subjects with similar characteristics) [28], the number of participants estimated to be necessary in each group to achieve a power of 80% and alpha = 0.05 was 18.

**Fig 2:**
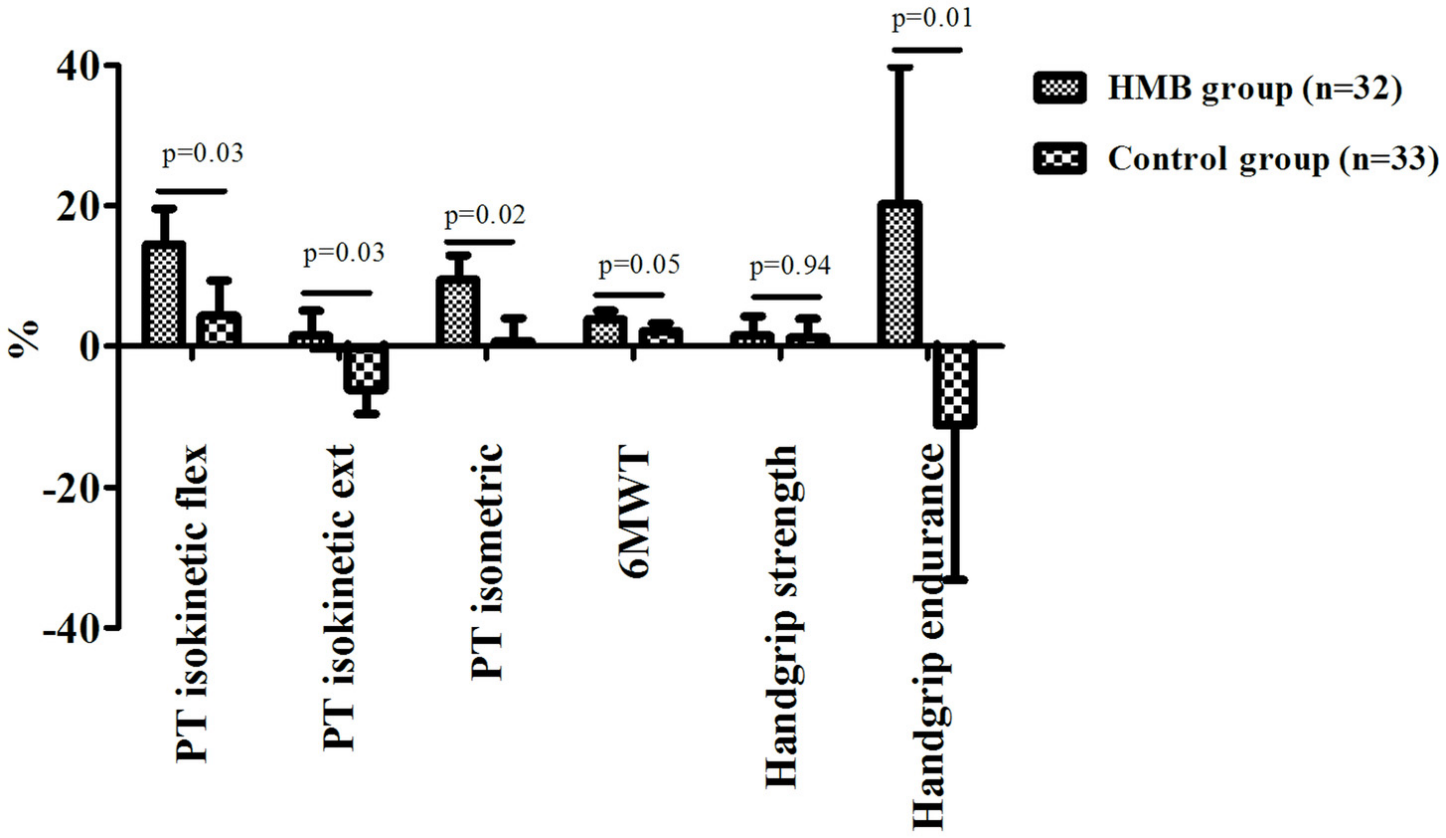
Changes in secondary outcomes (as percentages) from baseline to follow-up by group. Notes: the changes are represented as mean ± standard error of mean (SEM). Changes were calculated as the difference between the follow-up and baseline values (delta), divided by the baseline value and multiplied by 100. Abbreviations: PT = peak torque; 6MWT = 6-minute walking test.

For continuous variables, all parameters are presented as mean±SD (standard deviation), except for Fig 2, where results are given as mean±SEM for graphical reasons. The baseline characteristics of the treatment and control groups were compared using independent t-tests or chi-square tests, as appropriate. Paired t-tests were used for within-group comparisons of data recorded at the baseline and after 8 weeks, and changes were calculated as the difference between the two values (delta) A generalized linear model (GLM) was used for comparing the changes in primary and secondary outcomes at 8 weeks (dependent variable) between the two groups (independent variable), adjusted for the baseline value of the corresponding test (covariate) and without other adjustments for multiple testing. Due to the large number of variables assessed and the small sample size included, we considered the analyses of secondary outcome as exploratory. Significance was assumed if *p*≤0.05 (in the GLM we considered the p of the F test from model) and all tests were two-tailed. All analyses were performed using the SPSS 21.0 for Windows (SPSS Inc., Chicago, Illinois).

## Results

### Participants

The 80 eligible participants were randomized into two groups of 40 women each. Sixty-five participants completed the study, 32 in the treated group and 33 in the control group (Fig 1). The sample as a whole was a mean 69.5±5.3 years of age, with a mean weight of 64.7±11.4 kg, and a mean BMI of 26.3±4.2 kg/m^2^. Fifteen participants (7 in the control and 8 in the treated group) were lost during the follow-up: these women were similar to the sample as a whole in terms of age, baseline physical performance and strength, body composition, and levels of physical activity (details not shown).

Comparing the two groups, the treated participants were slightly older than the controls (71.0±5.3 vs. 68.2±4.5 years, respectively; *p* = 0.06), while the two groups did not differ in daily calorie intake (1621.29±396.98 vs. 1484.02±376.29 Kcal; *p* = 0.15), or protein intake (1.06±0.37 vs. 1.00±0.23 g/kg ideal weight; *p* = 0.43). The two groups’ physical activity levels, investigated with the PASE, were also similar, both at the baseline (193.9±40.8 in the treated group vs. 190.2±50.3 points in the control group; *p* = 0.75) and after 8 weeks (188.6±41.2 vs. 180.0±40.2 points in the treated and control groups, respectively; *p* = 0.37).

### Compliance

Based on bottle counts and specific questioning during the study (at 4 weeks and at the follow-up visit), the proportion of prescribed HMB doses probably used by the treatment group was 96±6%.

### Primary outcome

As shown in Table 1, there were no differences between the two groups’ total SPPB scores or balance test scores, walking times or chair stand times, neither at the baseline nor in the difference in changes of this outcome after 8 weeks.

**Table 1:** Participants’ mean baseline characteristics and outcomes at follow-up, by group.

	HMB-treated group (n = 32)			Control group (n = 33)			Between groups (change 8 weeks vs. baseline)	P value between groups
	Baseline^a^	8 weeks	Delta	Baseline^a^	8 weeks	Delta	Delta	
Measure	Mean±SD	Mean±SD	Mean±SD	Mean±SD	Mean±SD	Mean±SD	Mean±SD	
***Primary outcome***								
**SPPB,** total score	11.10±1.19	11.47±0.82	0.37±0.96	11.06±1.08	11.49±0.78	0.43±1.11	-0.02±0.19	0.92
**Balance test,** score	3.90±0.31	3.90±0.31	0.00±0.37	3.97±0.17	3.86±0.43	-0.11±0.47	-0.05±0.10	0.57
**Chair stand times**, s	10.62±2.67	9.29±2.21	-1.33±1.67	11.52±2.51	9.44±1.84	-2.08±1.76	0.24±0.36	0.51
**Walking time**, s	2.89±0.50	2.66±0.40	-0.24±0.30	2.89±0.40	2.66±0.33	-0.23±0.35	0.005±0.07	0.94
***Secondary outcomes***								
**PT isokinetic flex**, Nm	25.13±8.49	27.57±9.06	2.43±2.29**	25.13±7.33	26.10±9.06	0.97±5.54	1.56±1.56	0.03
**PT isokinetic ext**, Nm	55.73±16.72	55.77±16.06	0.03±6.16	54.23±12.61	50.94±16.44	-3.29±6.13	3.32±2.61	0.03
**PT isometric**, Nm	75.50±18.92	83.07±26.95	7.57±16.78**	77.00±21.32	76.35±22.79	-0.65±12.61	9.74±3.90	0.02
**6MWT,** m	498.72±62.58	518.40±63.93	19.68±20.21***	511.71±66.32	524.11±61.80	12.40±11.41	7.67±8.29	0.04
**Handgrip strength**, kg	18.90±4.14	19.35±4.57	0.46±2.57	21.71±5.06	21.36±4.88	-0.35±3.28	0.71±0.79	0.69
**Handgrip endurance,** s	74.65±65.13	89.66±57.24	15.01±41.36**	93.46±60.83	82.95±66.42	-10.52±45.16*	21.41±16.28	0.02
***DXA***								
**Weight**, kg	63.76±12.10	64.20±12.06	0.44±0.98	65.44±10.93	65.45±10.78	0.007±0.80	0.33±0.23	0.15
**Fat-free mass**, kg	38.36±4.84	38.96±5.13	0.33±0.83	38.38±5.83	38.57±5.68	0.19±0.94	0.12±0.24	0.69
**ASMMI**, kg/m^2^	6.39±0.79	6.42±0.80	0.03±0.25	6.44±0.73	6.33±0.77	-0.11±0.46	0.11±0.10	0.27
**Abdominal fat mass,** kg	10.82±3.87	10.89±3.93	0.07±0.53	11.70±3.63	11.60±3.43	-0.10±0.62	0.13±0.14	0.36
***Radial pQCT***								
**Muscle density,** mg/cm^3^	73.93±3.82	74.47±3.22	0.54±1.51*	73.98±3.53	73.66±3.40	-0.33±1.65	0.67±0.72	0.03
**Muscle area,** mm^2^	2577.46±401.55	2632.47±413.36	55.00±143.61	2624.98±428.42	2642.02±367.99	17.04±142.21	27.95±35.25	0.43
**Fat area,** mm^2^	1414.44±780.08	1395.07±708.00	-19.38±155.84	1505.43±678.46	1514.63±649.30	9.19±190.58	-9.19±41.48	0.20
**Fat/muscle ratio,** %	55.59±30.35	53.88±27.92	-1.70±6.77	58.44±27.60	57.98±26.29	0.46±9.50	-2.24±2.02	0.07
***Tibial pQCT***								
**Muscle density,** mg/cm^3^	71.31±5.58	71.99±3.62	0.68±1.08*	73.04±2.89	73.11±3.29	0.08±1.05	0.56±0.62	0.03
**Muscle area,** mm^2^	5799.47±798.20	5759.62±850.77	-39.85±426.20	6208.51±756.98	6169.35±728.08	-39.16±234.34	-0.02±93.00	0.86
**Fat area,** mm^2^	3204.93±1787.29	3592.97±2182.15	388.03±1695.72	3236.45±1942.34	3290.46±2071.02	54.01±276.73	301.02±321.42	0.35
**Fat/muscle ratio,** %	55.35±28.98	58.22±32.85	2.88±10.54	52.79±31.84	54.25±36.86	1.46±7.90	0.89±2.34	0.70

**Notes:**

^**a**^There were no statistically significant differences between the groups in any of the tests at the baseline.

Delta were calculated as the differences between data obtained at the baseline and at the follow-up after 8 weeks (within-group comparisons) using paired t-tests: p-values for these comparisons are given as:

*:p<0.05;

** p<0.01;

*** p<0.001.

A generalized linear model was used for a between-groups comparison of the changes obtained after 8 weeks, adjusted for the baseline value of the corresponding tests.

**Abbreviations**: SPPB: Short Physical Performance Battery; PT: peak torque; DXA: dual-energy X-ray absorptiometry; 6MWT: 6-minute walking test; ASMMI: Appendicular Skeletal Muscular Mass Index; pQCT: peripheral quantitative computerized tomography.

### Secondary outcomes

There were no differences between the groups at the baseline in terms of the secondary outcome measures investigated. After 8 weeks, the treated group performed significantly better than the control group in terms of PT isokinetic flexion (delta = 1.56±1.56 Nm; *p* = 0.03), PT isokinetic extension (delta = 3.32±2.61 Nm; *p* = 0.03), PT isometric strength (delta = 9.74±3.90 Nm; *p* = 0.02), 6MWT (delta = 7.67±8.29 m; *p* = 0.04), and handgrip endurance (delta = 21.41±16.28 s; *p* = 0.02), while no differences emerged for handgrip strength (Table 1). Fig 2 shows the changes (expressed as percentages) recorded in the two groups from the baseline to the follow-up. The greatest benefit in the treated group, compared with the controls, concerned handgrip endurance (20.12±19.56 vs. -11.18±22.04%, *p* = 0.01), followed by PT isokinetic flexion strength (14.55±5.1 vs. 4.36±4.98%, *p* = 0.03).

### Body composition parameters

1. **DXA.** There were no significant differences in weight, fat-free mass, abdominal fat mass or ASMMI between the treated and control groups, neither at the baseline nor in the difference in changes between baseline and 8 weeks of treatment (Table 1).
2. **pQCT.** No differences emerged between the two groups’ pQCT parameters for the radius and tibia at the baseline. After 8 weeks of HMB supplementation, however, the treatment group’s muscle density was significantly greater at both sites investigated (radius: delta = 0.67±0.72 mg/cm^3^; *p* = 0.03; tibia: delta = 0.56±0.62 mg/cm^3^; *p* = 0.03) than in the controls, while the other parameters considered were not significantly affected.

### Adverse events

No severe adverse events were reported in either group. Mild adverse effects of HMB supplementation included two cases of abdominal pain, two of constipation and one of itching, which regressed spontaneously after suspending the treatment.

## Discussion

This study investigated the effect of oral HMB supplementation on physical performance, muscle strength and body composition parameters in a group of healthy older women attending a twice-weekly mild fitness program.

Concerning our primary outcome, we found no significant differences between our treated and control groups in total SPPB score or any of the single items tested in this battery. There may be several reasons to explain these findings. First, it may be that the follow-up was not long enough to detect any improvement in SPPB score, which is a more complex measure than those assessing muscle power. Having said that, we feel that our short follow-up probably improved our participants’ compliance with the treatment. Second, our participants had high baseline scores for the SPPB, gait speed and chair stands time (probably because they exercised regularly), and this may have made any further improvement impossible. This ceiling effect seems to be particularly relevant for an item like the SPPB score, which is by nature categorical and consequently less sensitive to changes than a continuous numerical scale. [29] Analyzing gait speed and chair stands time as continuous variables did not substantially modify our results, however, suggesting that the SPPB is less sensitive than other measures of physical performance and muscle strength. Finally, it may be that the effects of HMB supplementation induce significant differences in measures that test muscle mass changes rather than muscle contraction speed, leading to significant differences in muscle density and muscle strength tests, but not in SPPB total or single-item scores. [29] It is also worth adding that our findings coincide with those of another study on healthy older people with high baseline SPPB scores. [30] Like ourselves, Stout et al. reported finding no significant improvements in healthy older men and women in the get-up-and-go test (which has similar characteristics to the SPPB). [31] Further research is needed on this aspect because the SPPB is an important predictor of various outcomes in older people. [32,33]

As for the secondary outcomes investigated, isometric and isokinetic muscle strength are important in the elderly: low levels of isokinetic and isometric strength are significantly associated with various negative outcomes in older women, such as falls, low bone mineral density, hip fractures, and functional limitations in activities of daily living. [34] Our study shows that an oral supplement containing HMB was able to improve both isometric and isokinetic muscle strength in active older women attending a mild fitness program. It is worth noting, however, that both these outcomes dropped somewhat in the control group at the follow-up by comparison with the baseline assessment, probably because the fitness program focused mainly on improving muscle contraction speed. Our results are consistent with other studies in which HMB was administered together with intensive resistance training (a type of exercise that would suffice alone to improve these parameters). [10] Although our study design (with no group taking a placebo) may limit their generalizability, our findings could be of clinical importance in the longer term because HMB supplementation might help to slow the physiological decline in muscle strength (estimated to occur at a rate of about 10–15% per decade). [34] On the other hand, we found no significant improvement in handgrip strength after HMB supplementation—a result consistent with another study reporting a substantial increase in the strength of the lower limbs, but not of handgrip. [12] Further studies are needed to clarify these results, given the importance of handgrip strength as a reliable parameter of several negative outcomes in the elderly. [35,36]

Albeit with the limitations due to it being an open-label trial, another important finding of our study is that HMB supplementation was able to improve handgrip endurance and 6MWT as well. Most of the activities of daily living demand a submaximal force [20], so the ability of oral HMB supplementation to improve these two items could be clinically important in delaying disability in older people.

The significant improvements observed in isometric and isokinetic strength, as well as in 6MWT and handgrip endurance, were associated not with any increase in muscle mass (as assessed by DXA), but with an increase in muscle density (investigated using pQCT). This morphological change might reflect a lower quantity of intramuscular fat and could be seen as an indication of better muscle quality. [37]

In addition, the significant improvements that we identified were obtained with lower doses of HMB than those used in other studies in older people. [10] Most researchers seem to agree that the optimal dosage of HMB is 3 g a day delivered in 3 equal doses. While this strategy might be appropriate in other conditions affecting older people (such as wasting diseases, loss of appetite, or gastrointestinal diseases), it might interfere with compliance in older people who are fit. [38] Moreover, HMB is an amino acid precursor and several works have shown that an excessively high protein intake is associated with a decline in renal function [39], so it may be that higher doses of HMB have a paradoxical effect on strength and physical performance. Be that as it may, the results that we obtained with a single daily intake of a low dose of HMB are encouraging and warrant further studies on healthy older people with a longer follow-up.

The present study has some limitations that need to be mentioned. First, it was not a double-blind trial, so we cannot rule out the possibility of the differences emerging between the two groups being due partly to the treatment group’s expectations. We also identified only small changes in muscle strength and physical performance parameters that, though statistically significant, might well be clinically irrelevant; this was probably due to the short follow-up considered. A second limitation lies in that only women were involved, so our findings may not be applicable to men too. A part of the literature suggests, however, that women experience a more severe muscle loss than men [40], and they are more liable to protein malnutrition. [41] Third, only women regularly attending a fitness program twice a week were included, and it may be that different results would emerge in other settings. Moreover, they are volunteers and also this aspect could add a bias in the interpretation of our findings. Another methodological limitation is that the changes between follow-up and baseline have a non-normal distribution probably due to the limited sample size included. Although GLM is fairly robust also for non-normal variables, the results should be interpreted with this consideration in mind. Another possible methodological limit could be considered the number of statistical tests performed that should be carefully considered in interpreting our findings. Finally, plasma or urinary HMB levels were not measured as markers of compliance in our study, although we did assess compliance frequently by means of weekly phone calls and a face-to-face interview during the study. A strength of our work, on the other hand, concerns the global assessment of physical performance and muscle strength, explored using reliable tests, such as DXA for body composition (the preferred method for assessing body composition in older people) [42] and pQCT, which enables muscle composition to be estimated too.

In conclusion, a nutritional supplement containing 1.5 g of calcium HMB for 8 weeks in our sample of healthy elderly women attending a twice-weekly mild fitness program had no significant effects on their total or single-item SPPB scores, but it did significantly improve several muscle strength and physical performance parameters. These findings suggest a role for HMB supplementation (at lower doses than those previously considered optimal) for fit elderly people too, with a view to preventing or delaying the age-related decline in some physical performance parameters. Further research is needed and should also compare the SPPB with other physical performance parameters.

## Supporting Information

S1 File Protocollo.docx: Original protocol approved by our IRB. (DOCX)

S2 File Study protocol. Pdf: Protocol (translated in English).
(PDF)

S1 Table CONSORT checklist. (DOC)
