# Supplementary material for: Effect of Oral Beta-Hydroxy-Beta-Methylbutyrate (HMB) Supplementation on Physical Performance in Healthy Old Women Over 65 Years: An Open Label Randomized Controlled Trial
Source: PLoS One. 2015 Nov 3;10(11):e0141757. doi: 10.1371/journal.pone.0141757 (PMC4631374; doi:10.1371/journal.pone.0141757)
Supplement: S1 File — (DOCX) [file pone.0141757.s001.docx]

PROTOCOLLO DI STUDIO

1. Titolo dello studio: “RUOLO DELLA SUPPLEMENTAZIONE ORALE DI PROTEINE E HMB NELL’ANZIANO IN RELAZIONE ALLA FORZA MUSCOLARE E ALLA PERFORMANCE MOTORIA”
   1. **Versione aggiornata al 08.01.2014**
2. **ISTITUZIONE PROPONENTE IL PROGETTO:**

ULSS 16 – U.O.C. Clinica Geriatrica.

- 1. **Coordinatore Scientifico:** Dott. Giuseppe Sergi, Dirigente Medico Geriatra, ULSS 16.
  2. **Gruppo di lavoro:** Dott.ssa A. Coin**,** Dott. F. Bolzetta, Dott.ssa M. De Rui, Dott.ssa L. Berton, dott. N. Veronese; dott.ssa S. Carraro; dott.ssa G. Bano; dott.ssa E. Valmorbida; dott.ssa G. Girotti. Dietista: sig.ra F. Miotto.

1. **INTRODUZIONE E RAZIONALE**

La sarcopenia è definita come la perdita di massa muscolare nell’anziano che porta inevitabilmente a perdita di funzionalità. Quest’ultima è a sua volta il più importante predittore di ospedalizzazione, cadute, fratture e mortalità nell’anziano. Un mantenimento più prolungato della massa muscolare appare quindi cruciale al fine di mantenere la funzionalità motoria e strumentale nell’anziano portando ad un miglioramento della qualità di vita. Ci sono evidenze che un aumento dell’intake proteico se associato ad attività sportive di resistenza può prolungare il mantenimento della massa muscolare. Tuttavia le informazioni riguardanti le necessità di intake proteico nell’anziano sono limitate. Ciò che emerge dall’attuale letteratura è che gli attuali valori indicate come idonei per la popolazione adulta non sono sufficienti a garantire il bisogno proteico della popolazione anziana. Si stima che più del 30% della popolazione anziana non consumi un quantitativo adeguato di proteine; questo nel tempo porta ad una diminuzione del turnover proteico dell’organismo con accelerazione della perdita di massa muscolare. L’attuale quantità di proteine raccomandata nella dieta di un adulto sano al di sopra dei 19 anni è di 0.8 grammi · kg^−1^ · d^−1^.

In particolare è stato dimostrato che un metabolita derivato dalla leucina, il beta-idrossi-betametilbutirrato (HMB) ha un effetto anticatabolico sulle proteine. Questa scoperta ha fatto si che tale metabolita sia divenuto di grande interesse come supplemento all’attività sportiva e nelle condizioni che inducono atrofia muscolare quali ad esempio la cachessia.

Scopo dello studio è quello di indagare se la supplementazione orale di proteine, ed in particolare di HMB, possa incrementare la forza muscolare nel soggetto anziano in buona salute.

1. **OBIETTIVI DELLO STUDIO**
   1. **Obiettivo primario**

Valutare le variazioni di performance fisica tramite Short Physical Performance Battery (SPPB) di un gruppo di anziane non sarcopeniche al tempo zero e dopo tre mesi di integrazione proteica rispetto un gruppo simile di anziani cui vengono forniti solo consigli dietetici.

- 1. **Obiettivi secondari:**

Valutare:

- - le variazioni della forza muscolare dell’avambraccio (handgrip).
  - le variazioni della forza muscolare nel movimento di abduzione del segmento tibiale (estensione).
  - le variazioni della capacità aerobica valutata con il 6 minute walking test (6MWT)
  - l’eventuale incremento di massa magra (FFM) rispetto al baseline con DXA e con pQCT.

**4.3 Variabili di esito**

La variabile risposta primaria è la variazione di performance fisica valutata con SPPB. Variabili di esito secondarie sono: la forza muscolare valutata a livello della mano nel movimento di adduzione delle dita (handgrip),  la forza muscolare delle gambe nel movimento di abduzione del segmento tibiale (estensione) e la FFM.

1. **DISEGNO DELLO STUDIO**
   1. **Durata dello studio:** Comprensiva di tutte le fasi: reclutamento, trattamento, follow-up, il progetto ha una durata di sei mesi. La parte sperimentale (trattamento e successivo controllo) avrà durata tre mesi. Il progetto partirà dal 01 marzo 2014.
   2. **Soggetti:**

*Criteri di inclusione:*

- Donne anziane non istituzionalizzate di età maggiore di 65 anni;
- autosufficienti nelle ADL
- MMSE >23
- Non sarcopeniche (secondo i criteri di Baumgartner)

*Criteri di esclusione:*

- Pazienti che già assumono integratori proteici per os.
- Pazienti con insufficienza renale (creatinina clearance < 30 ml/min.).

**Descrizione delle procedure di arruolamento:** Le pazienti eleggibili per lo studio saranno reclutate tramite l’ambulatorio nutrizionale della U.O.C Clinica Geriatrica, ULSS 16 e tramite contatto diretto con le palestre comunali afferenti alle “Attività creative della terza età”. Le partecipanti saranno divise in due gruppi. Al gruppo trattato (gruppo A) verrà somministrato un flacone di Ensure Plus Advance (vedi scheda tecnica) al giorno os mentre al gruppo di controllo (gruppo B) verranno forniti solo consigli dietetici riguardanti una dieta varia e completa dei fabbisogni giornalieri di proteine. La compliance e gli eventi avversi saranno seguiti telefonicamente settimanalmente e di persona dai medici dopo un mese di supplementazione.

- 1. **Numerosità campionaria**: Tenuto conto che l’obiettivo primario riguarda un confronto della performance fisica con SPPB nei soggetti che seguono i due programmi alternativi di trattamento, la numerosità campionaria è stata calcolata sulla base della variabile SPPB. Il confronto sarà effettuato sulla base della differenza in punti tra la il valore a tre mesi rispetto al basale, la cui distribuzione potrà essere approssimata ad una normale per una numerosità sufficientemente elevata. Un nostro precedente studio riguardo partecipanti in simili condizioni e per un periodo simile ha dimostrato che una numerosità di 18 persone per gruppo è necessaria per valutare una differenza tra i due gruppi di 1±1.51 punti tra i due gruppi con una potenza del 80% e con un α=0.05. Considerando un tasso di drop-out durante lo studio del 20%, una popolazione di 44 persone (22 per gruppo) sarà necessaria per verificare questo outcome. Questa numerosità sarà in grado di evidenziare come significativa una differenza di 4.0±5.6 kg nel movimento di adduzione delle dita (handgrip), di 5±5% nel movimento di abduzione del segmento tibiale (estensione), di 1.1±1.5 Kg per la FFM valutata con DXA e con pQCT e di 70±150 m nel 6MWT.
  2. **Analisi statistica:** I pazienti dei due gruppi saranno descritti mediante frequenze, per le variabili categoriali, e mediante media, deviazione standard e alcuni quantili selezionati per le variabili quantitative. La variazione delle variabili quantitative tra i due tempi (3 mesi-basale) sarà espressa mediante intervallo di confidenza dal 95%. I risultati saranno presentati per gruppo di trattamento. La significatività della differenza della variazione tra i due gruppi di soggetti sarà valutata mediante test t di Student per campioni indipendenti o il test non parametrico di Wilcoxon-Mann Whitney, qualora la distribuzione della variabile sotto esame fosse lontana dalla normalità.
  3. **Schema dello studio (flow chart):** In tutte le pazienti saranno eseguiti alla valutazione basale e dopo tre mesi i seguenti esami bioumorali e strumentali:
- Anamnesi ed inchiesta dietetica
- Indici antropometrici: peso, altezza, Body Mass Index (BMI).
- Composizione corporea (massa magra e massa adiposa) totale e distrettuale, mediante densitometria a doppio raggio X (DEXA) “total body”. Dalla somma della massa magra dei quattro arti, priva della massa ossea, sarà calcolata la massa muscolare appendicolare scheletrica per valutare la presenza di sarcopenia.
- Composizione delle componenti muscolari ed ossee mediante pQCT
- Misura della forza muscolare mediante Dynatronic 100 a livello degli arti superiori e inferiori. In particolare viene misurata la forza della mano nel movimento di adduzione delle dita (hand-grip) e delle gambe nel movimento di abduzione del segmento tibiale (estensione).

| ***FLOW CHART*** | ***CONTROLLO BASALE*** | ***CONTROLLO A TRE MESI*** |
| --- | --- | --- |
| - *Raccolta delle informazioni anagrafiche ed anamnestiche.* - *Inchiesta dietetica* - *Valutazione scale funzionali* - *Questionario PASE sull’attività fisica.* - *Antropometria: peso, altezza, calcolo del BMI.* - *Short Physical Performance Battery (SPPB)* - *Misurazione della forza muscolare con dinamometro* - *6 MWT* - *Densitometria “total body”* - *pQCT* | *X*  *X*  *X*  *X*  *X*  *X*  *X*  *X* | *X*  *X*  *X*  *X*  *X*  *X*  *X*  *X* |

1. **RISCHI PER LE PAZIENTI**

Nell’ambito dello studio, i pazienti non saranno sottoposti ad indagini invasive tranne che per il prelievo ematico che è comunque una procedura di routine. La dose di radiazioni a cui si è esposti nell’esecuzione della densitometria ossea e total body è di inferiore a 3 m REM e l’esame dura circa 10 minuti.

1. **RISULTATI ATTESI**

Visto il possibile impatto positivo della supplementazione proteica nella funzionalità muscolare, ci attendiamo un miglioramento della performance fisica nel gruppo trattato con integratori rispetto ai valori basali ed al gruppo di controllo. In particolare, ci attendiamo una differenza significativa di 1 kg di forza muscolare misurata come handgrip dopo tre mesi tra i due gruppi esaminati. Tra gli obiettivi secondari ci aspettiamo un incremento della massa magra in entrambi i gruppi, ma in particolare nel gruppo trattato con l’alimento per uso specifico.

Visita ambulatoriale

(deXa, inchiesta, forza, esami)

Gruppo **A**

Gruppo **B**

Suggerimenti dietetici ed integrazione con Ensure plus

Suggerimenti dietetici

Valutazione Clinica

Al tempo 0 e dopo tre mesi con composizione corporea e prove di forza/performance

Valutazione Clinica

Al tempo 0 e dopo tre mesi con composizione corporea e prove di forza/performance

**BIBLIOGRAFIA**

1. Evans WJ. Protein nutrition, exercise and aging. J Am Coll Nutr. 2004 Dec;23(6 Suppl):601S-609S.

2. Eley HL, Russell ST, Baxter JH, Mukerji P, Tisdale MJ. Signaling pathways initiated by beta-hydroxy-beta-methylbutyrate to attenuate the depression of protein synthesis in skeletal muscle in response to cachectic stimuli. Am J Physiol Endocrinol Metab. 2007 Oct;293(4):E923-31. Epub 2007 Jul 3.

3. Malafarina V, Uriz-Otano F, Iniesta R, Gil-Guerrero L. Sarcopenia in the elderly: diagnosis, physiopathology and treatment. Maturitas. 2012 Feb;71(2):109-14. Epub 2011 Dec 6.

4. Baumgartner RN, Koehler KM et Al. Epidemiology of sarcopenia among the elderly in New Mexico. Am J Epidemiol. 1998 Apr 15;147(8):755-63.

5. Zanchi NE, Gerlinger-Romero F et Al. HMB supplementation: clinical and athletic performance-related effects and mechanisms of action. Amino Acids. 2011 Apr;40(4):1015-25. Epub 2010 Jul 6.
