# Supplementary material for: Effect of Oral Beta-Hydroxy-Beta-Methylbutyrate (HMB) Supplementation on Physical Performance in Healthy Old Women Over 65 Years: An Open Label Randomized Controlled Trial
Source: PLoS One. 2015 Nov 3;10(11):e0141757. doi: 10.1371/journal.pone.0141757 (PMC4631374; doi:10.1371/journal.pone.0141757)
Supplement: S2 File — Pdf: Protocol (translated in English). (PDF) [file pone.0141757.s002.pdf]

Role of Oral Supplementation of Proteins and β-hydroxy-β-methyl Butyrate (HMB) in a Sample of Older Women in Good Health

Purpose

The purpose of this study is to determine whether a supplementation containing β-Hydroxy-β-methylbutyrate is effective in the treatment of age-related muscle loss in a group of older women compared to a non-interventional group

Outcomes

Primary Outcome Measures:

- Change in Short Physical Performance Battery [Time Frame: Baseline and after 2 months]

Enrollment: 80

Study Start Date: February 2014

Study Completion Date: June 2014

Primary Completion Date: June 2014 (Final data collection date for primary outcome measure)

Eligibility

Inclusion Criteria:

- People over 65 years, not residents in nursing home, in good health
- Mini Mental State Examination (corrected for scholarity) more than 23

Exclusion Criteria:

- Previous oral supplementation with proteins or minerals
- Important co-mordidities, such renal or hepatic failure.

Characteristics of the groups

Arms	Assigned Interventions
Experimental group: HMB	Control group
Group taking oral supplementation with Beta-hydroxy-beta-methylbutyrate	No Intervention
